# The NeuroinflammatoryPotential of HIV-1 NefVariants in Modulating the Gene Expression Profile of Astrocytes

**DOI:** 10.3390/cells11203256

**Published:** 2022-10-17

**Authors:** Sushama Jadhav, Prajakta Makar, Vijay Nema

**Affiliations:** 1 Indian Council of Medical Research, Department of Molecular Biology, National AIDS Research Institute, 73, G Block, MIDC, Bhosari, P.O. Box No. 1895, Pune 411026, Maharashtra, India; 2 Faculty of Health Sciences, Symbiosis International (Deemed University), Lavale, Mulshi, Pune 412115, Maharashtra, India; 3 Institute of Bioinformatics and Biotechnology (IBB), S. Phule Pune University, Pune 411007, Maharashtra, India

**Keywords:** IHDS, HAND, neurotoxicity, RNA interference, neuroinflammation

## Abstract

HIV-1 mediated neurotoxicity is thought to be associated with HIV-1 viral proteins activating astrocytes and microglia by inducing inflammatory cytokines leading to the development of HIV-associated neurocognitive disorder (HAND). In the current study, we observe how HIV-1 Nef upregulates the levels of IL-6, IP-10, and TNF-α around 6.0fold in normal human astrocytes (NHAs) compared to cell and empty vector controls. Moderate downregulation in the expression profile of inflammatory cytokines was observed due to RNA interference. Furthermore, we determine the impact of inflammatory cytokines in the upregulation of kynurenine pathway metabolites, such as indoleamine 2,3-dioxygenase (IDO), and 3-hydroxyanthranilic acid oxygenase (HAAO) in NHA, and found the same to be 3.0- and 3.2-fold, respectively. Additionally, the variation in the level of nitric oxide before and after RNA interference was significant. The upregulated cytokines and pathway-specific metabolites could be linked with the neurotoxic potential of HIV-1 Nef. Thus, the downregulation in cytokines and kynurenine metabolites observed after siRNA-Nef interference indicates the possibility of combining the RNA interference approach with current antiretroviral therapy to prevent neurotoxicity development.

## 1. Introduction

HIV-1 infection is reported to be a public health problem globally. There were approximately 1.7 million new infections and 680 thousand deaths due to AIDS-related illnesses by the end of the year of 2020, while 37.7 million individuals are living with HIV infection [1]. Combined antiretroviral therapy (cART) is a standard therapy for HIV infection to help in the reduction in viral load and prolong life expectancy; however, viral genetic variability has been linked with an interruption in the normal progression of the disease or progression towards neurological complications [1,2,3]. Despite the availability of cART, there is a need for a safe, cost-effective, easily accessible vaccine to attain global control of the HIV pandemic; however, there are various hurdles in the development of an effective vaccine [4]. At present, with the availability of cART, an increase of HIV-1 viral copies in the brain compartment is linked with the development of HIV-associated neurocognitive disorders (HANDs) along with multiple physiologic and psychosocial factors [5]. Since 2005, the International HIV Dementia Scale (IHDS) has been extensively used as a screening tool to understand the prevalence of HAND [6]. Frascati criteria have also been defined as another tool that can be used to standardize the research criteria for the diagnosis of HAND [7]. Apart from this, neuronal cell death is frequently observed in the brains of HIV-1-positive individuals, irrespective of the ART regimen [8]. The direct effect of HIV-1 infection in the brain is the neurotoxicity that developed due to the virus [9] as well as due to virally encoded proteins, viz., the gp120 [10] transactivator of transcription (Tat) [11] and negative factor (Nef) [12].

Among the different accessory proteins, Nef is one of the most variable proteins of HIV-1 [13] and remains an important factor for developing AIDS and HAND [5,14]. Nef translates into a 27 to 35 kDa protein expressed in the initial phase of viral replication, and it is expressed by latently infected cells and astrocytes [15,16]. HIV Nef is highly neurotoxic, and Nef by itself is enough to yield oxidative stress, axon degeneration, and BBB damage [17]. Moreover, HIV Nef has numerous functions, particularly the enhancement of viral infectivity and viral replication [18] and the modulation of cell-surface receptors, namely, the downregulation of the CD4 receptor, [19] upregulation of CD74, [20] downregulation of HLA class I (HLA-I), and cellular signaling pathways affecting the intracellular organization. An affinity of predominant epitopes of viral proteins with the binding groove of HLA class I molecules along with other host factors, as well as environmental factors, can be a significant determinant for regulating a load of integrated DNA and the expression of viral genes leading to differential pathogenesis of neurological disorders [21,22]. Different roles of this protein are linked with disease development and are associated with several motifs, namely, N-Myristoylation, the proteolytic cleavage region, the proline-rich motif, the acidic region, the dileucine, and FPD motif [12]. The sequence diversity in many regions of the HIV-1 Nef protein could be associated with the disruption in the overall functioning of the protein and thus be responsible for altering disease progression and pathogenesis in HIV-1-infected hosts [23]. Moreover, the HIV viral protein Nef is reported to be responsible for activating astrocytes and microglia and releasing cytokines leading to HIV-mediated neuroinflammation and neurotoxicity along with associated behavioral changes. Various studies have been published on the effect of Nef on the transcription of viral and cellular genes in different types of cell lines [24].

Viral genetic diversity also plays an important role in the evolution of the virus. This can be determined by the phylogenetic analysis to find out the subtype of the viral strain for understanding HIV transmission dynamics and the viral evolution pattern for effective HIV control [25,26]. Moreover, HIV-1 genetic variability and subtype-specific signatures in accessory proteins significantly influence gene expression profiles developing neuropathogenesis in HIV-associated neurocognitive impairment [27].

In addition to the viral factors, tryptophan metabolites via the kynurenine pathway from the host (kynurenine and quinolinic acid) are also associated with neurocognitive impairment in the host HIV-positive individuals [28]. Furthermore, neurotoxicity caused by these metabolites shows an association with changes in behavior, sleep, body temperature, and mood swings [29]. The association of tryptophan metabolism with neurotoxicity is evidenced by the production of the nicotinamide adenine dinucleotide (NAD^+^) and other neuroactive intermediates following the kynurenine pathway. Most of the kynurenine-pathway intermediate metabolites, namely, kynurenine (KYN), kynurenic acid (KYNA), 3-hydroxykynurenine (3-HK), picolinic acid (PIC), and quinolinic acid (QUIN), are found to be induced in astrocytes, macrophages, and microglia due to cytokines triggered after their exposure to HIV viral proteins [30].

Furthermore, host genetic factors, namely, human leucocytes antigen class I (HLA) molecules, have an important role that is expressed on the surface of all nucleated cells. The intricate interaction of HLA class I molecules facilitates the activation of CD8+ T cells generating a cytotoxic T-lymphocyte (CTL) response while class II molecules help recognize antigen-presenting cells routing them to CD4+ T cells and facilitating the production of cytokines [31]. Thus, several factors play an important role in the development of HIV-associated neurotoxicity.

In the present study, we use normal human astrocytes (NHAs) to explore the effect of HIV Nef in patients with and without dementia on modulating the cytokine and kynurenine metabolite expression profiles, while primary human fetal astrocytes (PHFAs) were used by Liu et al., 2015 and Saribas et al., 2015 to study the differential expression of cytokines as well as the PI3K pathway components and to explore the effect of HIV Nef in CNS with respect to autophagy using an adenovirus vector (Ad-Nef), respectively. Furthermore, astrocytes are the active, dynamic signaling players of the brain compartment and brain disorders are usually characterized by the inflammatory state and dysfunctioning of the astrocytes. Reactive astrocytes play a major role as a defense mechanism to reduce and repair damage to the central nervous system (CNS). Moreover, targeting astrocytes could be an alternative way to design novel therapeutic strategies.

In the present study, we construct Nef plasmids using HIV strains from clinical samples with and without HAND and with known subtype-specific plasmids. The Nef genetic diversity of the clinical samples is determined by sequencing and the phylogenetic tree. We also predict HLA-binding epitopes for a consensus of the Nef sequence of the studied strains using ProPred-I HLAs binding-prediction software. Furthermore, we describe the potential of the HIV Nef virotoxin in stimulating the cytokines and pathway-specific metabolite-expression profile by transfecting Nef plasmids in normal human astrocytes (NHAs), and their inhibition due to RNA interference. The upregulated level of inflammatory cytokines (IL6, IP-10, and TNF-α) as well as kynurenine-specific metabolites (IDO and HAAO) in NHA is quantified by real-time PCR at the transcriptional level and further confirmed the intracellular expression of Nef virotoxin at the translational level by Western blot. The action of siRNA-Nef is studied to understand the downregulation in the expression profile of cytokines and metabolites at the transcriptional level. The cytokine expression level at the translational level is assessed by the Bio-Plex assay. As previously reported, in the inflamed brain, a high volume of nitric oxide is produced by microglia and astrocytes and this excessive nitric oxide causes neuronal toxicity and neuroinflammation. Nitric oxide (NO) was first characterized as an endothelium-derived relaxation factor and has now emerged as a ubiquitous signaling messenger molecule involved in diverse pathophysiological processes, such as neurotransmission and inflammatory and immune responses, and in excess can cause cell death. Thus, an increased level of nitric oxide is indicative of neurotoxicity. Hence, its level is determined by the Griess reagent before and after RNA interference from culture supernatants. It is evident from these results that Nef virotoxin triggers a neuroinflammatory profile and is partially inhibited due to siRNA-Nef interference as observed in the astrocytes fulfilling one of the objectives of this study.

Microglia and astrocytes produce high-volume nitric oxide during inflammation within the inflamed brain. Excessive nitric oxide causes neuronal toxicity and death, and mesenchymal stem cells can be used as an approach to limit the neuronal damage caused by neuroinflammation.

## 2. Materials and Methods

### 2.1. Details of the Study Samples

The Institutional Ethics Committee’s approval (IEC) was obtained before starting the proposed study. The approved protocol number of the Institutional Ethics Committee is NARI-EC/2015-12 Version 1.0, 25 April 2015. The samples were considered from a previously collected cohort and hence are not representative of the ART status at present. These HIV-positive samples were considered after delinking the patient identities, but inclusive of the parameters depicted in Table 1. The determination of neurocognitive impairment was performed by assessing at least seven areas of neurocognitive functioning that are known to be affected by HIV infection (fluency, executive functions, speed of information processing, motor skills, attention, learning, and memory) by using a performance-based comprehensive neurocognitive battery and interpreting this using a demographically appropriate normative process along with the history of ATT (anti-tubercular treatment) and ART.

### 2.2. Amplification and Genetic Analysis of HIV nef Gene Sequence

**Figure 1:**
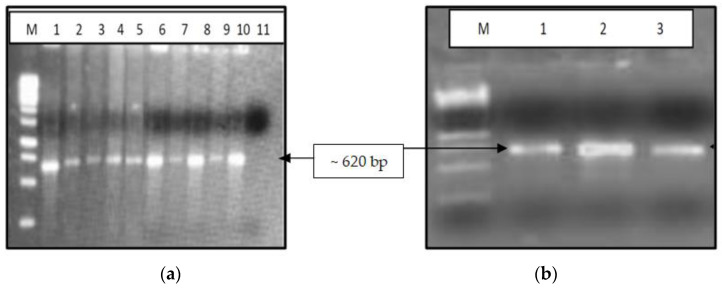
The 1.2% agarose gel electrophoresis of HIV-1 *nef* gene amplified using PCR. (**a**) Lane marked with M: 1 kb DNA ladder, lanes marked 1–10 = IHDS 1–10 clinical samples, 11 = negative control (**b**). Lane marked with M: 1 Kb DNA ladder, 1 = pIndieCNef, 2 = pNL4.3 Nef, 3 = pLconsNef).

**Figure 2:**
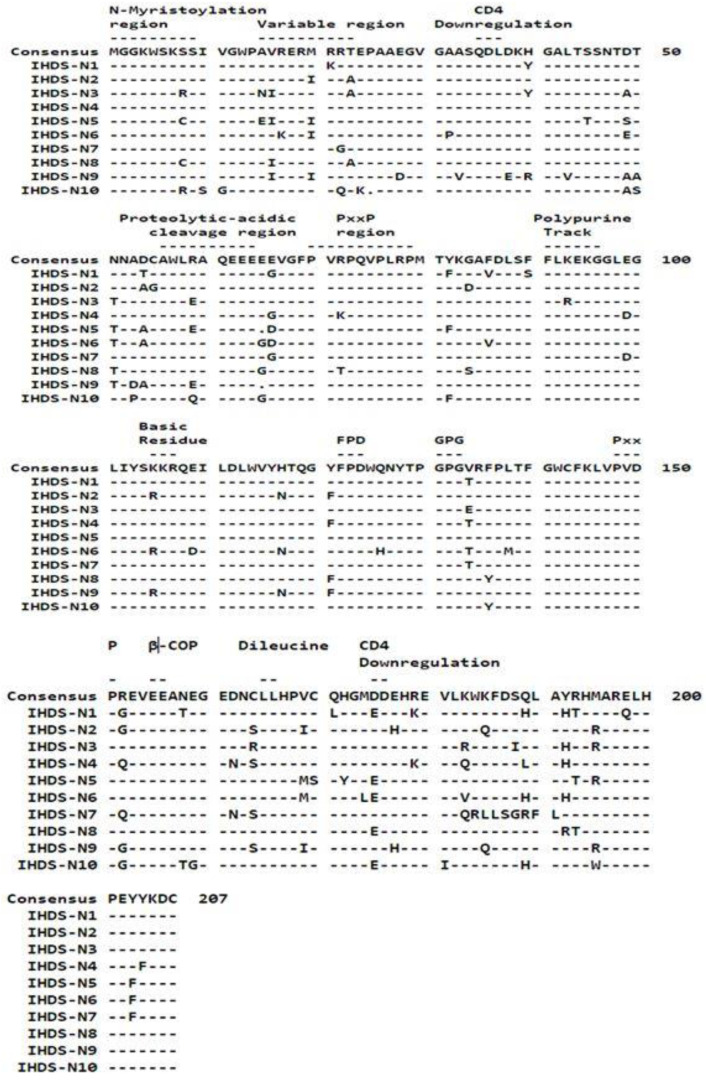
Amino acid alignment of HIV-1 Nef sequences with the consensus sequence developed from 10 study samples. Different regions of Nef sequences are indicated at the top of the consensus sequence.

The isolation of viral RNA was conducted using ten plasma samples at the Viral RNA Mini Kit (Qiagen, Studio City, CA, USA), followed by the synthesis of cDNA using a commercially available kit (first-strand synthesis system, Thermofisher Scientific, Inc., CA, USA) and nested PCR using full-length *nef* gene-specific primers to amplify a 620 bp fragment followed by its confirmation by gel electrophoresis. The primers used to amplify the HIV *nef* gene (8797-9414, HXB2) in the first round of PCR were N-1 5′- ATTAGAGTTAGGCAGGGATA-3′, N2 5′-CTGGTCTAACCAGAGAGACCCAGTAC-3′, while the second-round PCR primers wereN3 5′-AAGAATTCGGATGGGT/GGGCAAGTGGTCAA-3′ N4 5′-ATAAGAAGCGGCCGCTCAGCAGTCTTTGTA-3′ (Figure 1a,b). To determine the genetic variation in the *nef* gene, the confirmed HIV *nef* amplicons were sequenced using a genetic analyzer (ABI 3730, CA, USA) following Sanger sequencing technology. Genetic variability was determined by analyzing the sequences using SeqScape software (v 2.7), while MEGA X software was used to construct neighbor-joining trees using the Jukes–Cantor correction with 1000 replicates for the bootstrap analysis. Additionally, amino acid sequences were aligned using the seqpublish tool from the HIV database to generate the alignment and determine the variations in the amino acid sequences, as depicted in Figure 2. At the same time, we performed a subtype analysis of the clinical samples based on the phylogenetic tree to understand the clustering pattern of the study samples considering the published subtype-specific sequences obtained from different geographical locations. The consensus *nef* sequence of the study samples was subjected to analyzing the HLA epitopes using the software available online.

### 2.3. Cell Culture and Estimation of Cell Viability 

Normal human astrocytes (NHAs) as primary cells were obtained from Lonza Inc. (NHA, Cat No. 2565, Lonza Inc., Basel, Switzerland). Normal human astrocytes were cultured in Dulbecco’s Modified Eagle Medium (DMEM/F12, Gibco, Invitrogen, CA, USA Cat No. 31765-035) supplemented with 10% heat-inactivated HI fetal bovine serum (HI FBS, Cat No. 10082-147, Gibco, Invitrogen, CA, USA) at 37 °C and 5% CO_2_. NHA cells were maintained at 37 °C in 5% CO_2_ and 95% environmental air and 8–10 passages were maintained before using the cells for the actual experiment. The cells were tested for mycoplasma contamination before using the cells for the actual transfection experiment. The culture media were replaced every two days depending on the cell confluency.

The cytotoxicity of siRNA was estimated in normal human astrocytes (NHAs) using MTT (3-(4,5-dimethylthazol-2-yl)-2,5-diphenyltetrazolinum bromide, MTT (Sigma-Aldrich, Inc., St. Louis, MO, USA) reduction-based activity measured with a colorimeter. The cells were plated in 24-well plates (1 × 10^5^ cells/well), 24 h before the experiment. The cells were treated with different concentrations of siRNA (50, 100, 150, and 200 nM) in serum-free media, while untreated cells were considered as a control group. After following incubation, the medium was replaced and the cells were incubated further for 24 h. Cell viability was determined by adding 10 μL MTT (5 mg/mL) to each well. Following incubation, the MTT solution was removed and formazan crystals were solubilized in molecular-grade dimethyl sulfoxide (DMSO). The absorbance was measured at 570 nm using a microplate assay reader (Bio-Tek, Santa Clara, CA, USA) and a graph was plotted to understand the optimum concentration as compared to the control cells for further transfection experiments.

### 2.4. Construction of Plasmids for Transfection

The HIV *nef* gene obtained from study subjects along with HIV subtype-specific plasmids, namely, pIndieC for subtype C, pNL4.3 for subtype B, and consensus sequence of subtype C, was amplified for the *nef* gene. The plasmids pIndieC, pNL4.3, and pLconsNefSN were obtained under the NIH HIV Reagent Program, Division of AIDS, NIAID (NIH provided by Dr. Ron Swanstrom). The primers were designed with the cutting sites of restriction enzymes. The primers for amplification covering the entire *nef* (8797–9414, HXB2) were used with a high-fidelity enzyme (Perkin Elmer) and other necessary reagents, as previously mentioned [32]. The ligation reaction was performed to clone the purified amplicon into the pCMV-HA vector using the T4 DNA ligase followed by the transformation in the bacterial system to expand the plasmids of each *nef* gene for further experiments. Additionally, to determine the transfection efficiency, the pEGFP-N1 vector (plasmid-encoding enhanced green fluorescent protein where the protein of interest fused to its N terminus, Clonetech, CA, USA) was procured for cloning the *nef* gene and obtainapEGFP-N1-Nef plasmid construct and analyzing the transfection efficiency by fluorescence microscopy using a Keyence Biorevo BZ-9000 device armed with GFP-B excitation filters. Fluorescence images were recorded and processed by BZ-II viewer software (Osaka, Japan). The percentage of cells transfected was estimated based on fluorescence microscopy and flow cytometry.

### 2.5. Plasmid and siRNA-nef Transfection in NHA

The primary normal human astrocytes were seeded at a density of 5 × 10^5^ cells/well in 12-well plates and grown to obtain 80% confluence before starting the transfection experiment. Approximately 1 µg of Nef plasmids were added to the transfection reagent (Lipofectamine 3000) and incubated at room temperature for 5 min to form a complex in 500 µL of serum-free medium and transfect the cells as per the manufacturer’s instructions (Invitrogen Inc., Carlsbad, CA, USA). Subsequently, the transfection media was replaced by a freshly prepared medium with 10% FBS. In separate experiments, 5 × 10^5^ cells in each well were transfected with 50 nM HIV siRNA-Nef targeted to *nef* sequence (GTGCCTGGCTAGAAGCACA) in serum-free media in 12-well plates. HIV siRNA-Nef was added 48 h before transfection with HIV-1 Nef plasmids of clinical samples and subtype-specific strains. After 24 h, the transfection medium was replaced with fresh DMEM supplemented with 10% FBS. Astrocytes were trypsinized and seeded at a density of 5 × 10^5^ per well in 12-well plates. Nef transfection was then performed the next day as previously performed, and the cells were harvested for the estimation of cytokines together with RNA and protein levels. Transfection with 50 Nm siRNA-Nef along with scrambled siRNA-Nef were used as a negative-control RNA. The cultured cells were incubated in a CO_2_ incubator at 37 °C and harvested for the extraction of RNA or protein.

### 2.6. Real-Time RT-PCR and Multiplex Cytokine Assay

Using cytokine-specific reverse primers, isolated RNAs were converted to cDNA using the SuperScript III first-strand synthesis system (Thermofisher Scientific, CA, USA). Quantitative real-time reverse transcriptase-polymerase chain reactions (q RT-PCR) were conducted using a 7900HTReal-Time PCR system (Applied Biosystems Inc.) to estimate the mRNA expression levels using SYBR green chemistry. The reaction conditions included reverse transcription at 50 °C for 2 min, denaturation at 95 °C for 10 min, followed by amplification for 40 cycles (95 °C for 15 s, 60 °C for 1 min). The IL-6, IP-10, TNF-α, IDO, KMO, and HAAO primers were used for quantitative estimations. GAPDH was used as a housekeeping gene to normalize the expression of all genes using the 2^(−ΔΔCT)^ method. Primers were synthesized with IDT technology, as previously published, and accordingly optimized annealing temperatures. Subsequently, the culture supernatants were considered for the estimation of the cytokine expression profile before and after RNA interference using HIV siRNA-Nef to evaluate the change in the expression level of the cytokines. A Bio-PlexPro^TM^ Human cytokine Group I Panel 17-Plex assay was used to examine the level of cytokines obtained from NHA and quantified using Bio-plex manager software v 5.0 along with the 5PL standard curve.

### 2.7. Western Blotting

Following the transfection experiment, the cells were lysed in lysis buffer (150 mM sodium chloride, 1.0% Triton X-100, 50 mM Tris pH 8.0) inclusive of a 1 X protease inhibitor cocktail (Hi Media Laboratories, India), and around 25 µg of cell lysates were loaded on 12% SDS-PAGE gel for resolving protein bands at 100 volts for 2 h maintaining the temperature of the running buffer. The proteins were transferred to the PVDF membrane using the semi-dry (SD) Trans-Blot apparatus (Biorad Inc., CA, USA) without exceeding a voltage of 25 volts. The blots were then blocked in 5% nonfat dry milk for 1 h to avoid non-specific binding in TBST buffer (20 mM Tris-buffered saline (pH 7.5) with 0.1% Tween20) followed by overnight incubation with anti-HA-tagged antibody produced in rabbits (Cat. No. 26183, Dilution 1:3000, Thermofisher Scientific Inc., Waltham, MA, USA). The following day, the blots were incubated for 2 h in goat anti-rabbit IgG (H+L) secondary antibody, HRP (Cat No. 31460, dilution 1: 100,000, Thermofisher Scientific Inc., Waltham, MA, USA). The blots were washed 5–6 times using TBST buffer containing 0.05% Tween 20 after incubation with primary as well as secondary antibodies to reduce non-specific binding. Proteins were visualized by adding super signal west dura extended duration substrate (Cat No. 37071, Thermofisher Scientific Inc., Waltham, MA, USA) using chemiluminescence detection (Chemidoc XRS, Bio-Rad, CA, USA).

### 2.8. Estimation of Nitric Oxide as a Biomarker of Oxidative Stress

The concentration of nitric oxide was measured in a standard Griess reagent provided by Hi media (EZ assay nitric oxide estimation kit). Briefly, 100 µL of culture supernatant was mixed with 100 µL of reducing agent (CCK061), and 50 µL of each Griess reagent I and II, followed by the incubation of the plate at 37 °C for 2–4 h, and the absorbance was read at 580 nm (main)/630 nm. As specified, dilutions of the sodium nitrate and sodium nitrite standard were performed to generate a standard calibration curve at different dilutions to achieve different concentrations (data not shown). The assay was performed in duplicate, and the means of the three assays were considered to plot a graph of estimated nitric oxide in a micromolar (µM) concentration with the control baseline supernatant as the blank.

### 2.9. Statistical Analyses

Statistical analysis included the means while standard errors were measured using GraphPad Prism software Version 8.0 (Graph pad San Diego, CA, USA). The quantification of kynurenine pathway metabolites and cytokine levels were expressed as mean + SEM. The histograms were plotted with the analysis of significance using the student’s unpaired *t*-test, ANOVA. A *p*-value of <0.05 was considered statistically significant.

## 3. Results

### 3.1. Study Participants

The demographic details of the HIV-positive study participants are depicted in Table 1 along with the International HIV Dementia Score (IHDS), CD4 count, viral copies, and clinical findings. The IHDS score for five samples was <9.5 and identified as with dementia, while for the other five samples the score was >9.5 and identified as without dementia. We amplified the *nef* gene of the viral strains from these plasma samples. Additionally, HIV-1 subtype-specific plasmids, namely, pIndieC for subtype C, pNL4.3 for subtype B, and HIV *nef* consensus sequence of pLconsNefSN for subtype C, were obtained from the NIH reference reagent repository, Bethesda, MD, USA.

### 3.2. HIV nef Amplification, Sequencing, and Plasmid Construction

The amplicons of all the ten clinical samples (IHDS 1–10) along with DNA from HIV-negative healthy controls and subtype-specific plasmids are depicted in Figure 1a,b.

**Figure 3:**
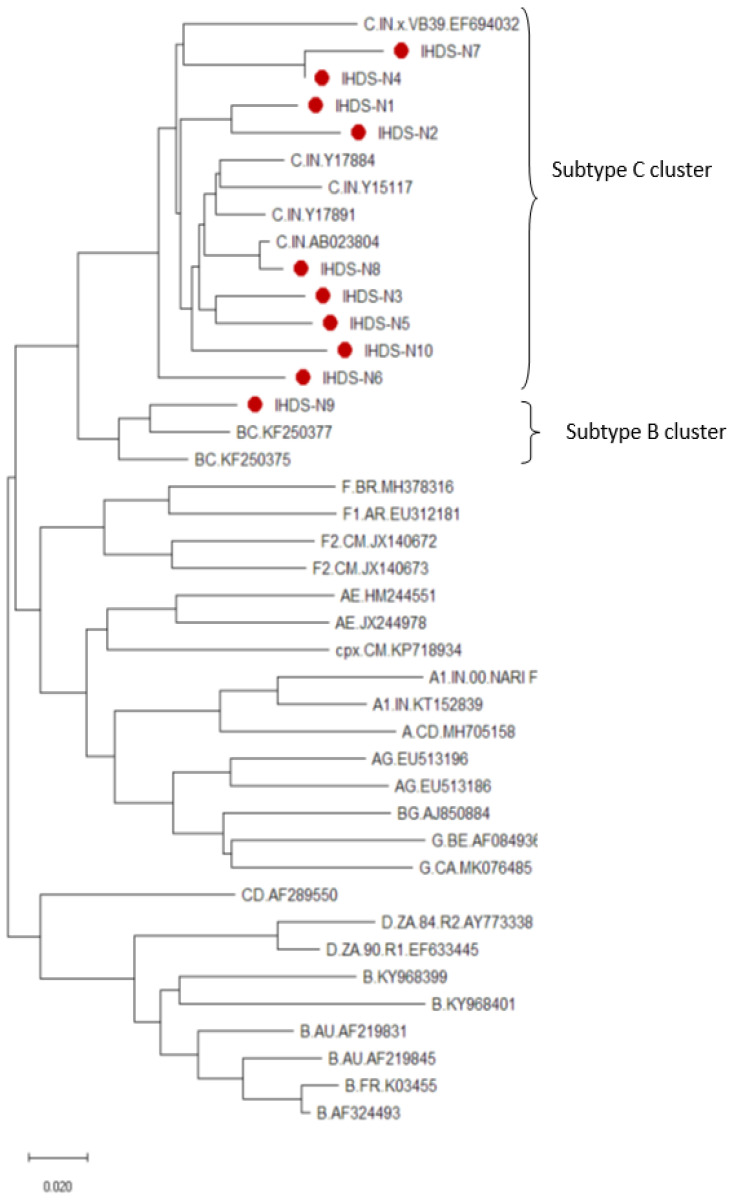
The phylogenetic tree of *nef* gene study sequences and other reported HIV-1 subtype-specific sequences along with consensus sequences. The subtypes cluster includes all the study samples, i.e., IHDS 1 to 8 and IHDS-10, while the subtype-B cluster includes the IHDS-9 sample. The horizontal branch length represents the evolutionary distance, and the vertical distance represents relatedness. The study sequences are marked with a red dot.

Purified amplicons of all the study samples were sequenced for understanding genetic variability and subtype identification by constructing an amino acid alignment using the seqpublish tool from the HIV database and a phylogenetic tree, respectively. The amino acid variations in the alignment are depicted in Figure 2 wherein samples 1–10 are equivalent to the Id No. of the samples as IHDS 1–10. The tree shows a formation of a cluster of subtype-C-specific sequences from different geographical regions with nine study samples in the same cluster indicating the predominance of subtype C, except for one sample forming a small cluster of BC recombinants (Figure 3). All these analyzed sequences were submitted into the GenBank database (accession numbers: MT337374-MT337383). The HIV *nef*-purified amplicons were cloned in the pCMV-HA vector to construct plasmids for the transfection of normal human astrocytes (NHAs).

### 3.3. Prediction of HLA-Binding Epitopes

**Figure 4:**
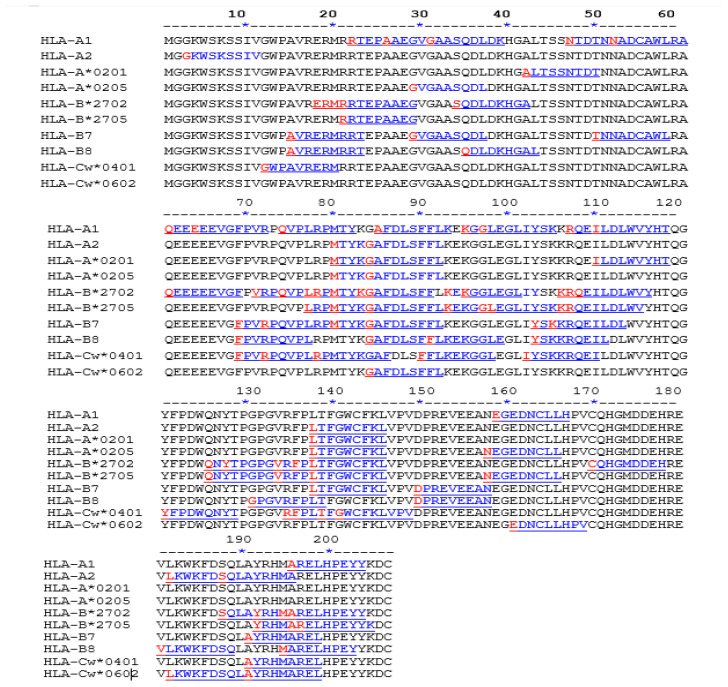
Deduced HLA-binding epitopes for the consensus *Nef* gene sequence of study strains of HIV-1. The 10 most common high-frequency HLA motifs predominant in the Indian population are included for prediction using ProPred-I HLAs binding-prediction software.

We predicted the HLA-binding and -non-binding epitopes considering the consensus Nef sequence of the study samples. The epitopes GAFDLSFFL (aa 83) and LTFGWCFKL (aa 138) were found to bind with HLA molecules at 60% and 80%, respectively. The RTEPAAEGV (aa 22) epitope was bound toHLA-A1 and non-binding to HLA-A2, while the MARELHPEY (aa 195) epitope bound to HLA-B8 and HLA-cw*0602 and non-binding to HLA-B7. Predicted binding epitopes of Nef to host proteins highlight their importance in modulating the virus–host interaction, as depicted in Figure 4.

### 3.4. Determination of Cytotoxicity of siRNA-Nef

**Figure 5:**
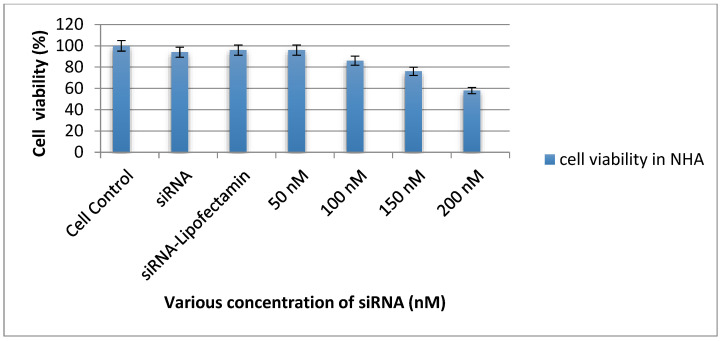
The cytotoxicity of siRNA is tested in NHA. The cell viability is estimated by MTT assay considering different concentrations of siRNA and cell control. The cells are transfected with transfection reagent forming siRNA complex with concentrations of 50, 100, 150, and 200 nM of siRNA. The percentage of cell viability is the means ± SD of two different experiments.

Various concentrations of siRNA-Nef (50 nM to 200 nM) were tested to determine the cell viability of astrocytes and we observed that 50 nM of concentration was non-toxic in comparison with the untransfected cell control and scrambled siRNA and a higher concentration of siRNA-Nef. It was observed that with increasing the concentration of siRNA-Nef, the viability of cells was reduced in both types of cells (*p* < 0.01), as depicted in Figure 5.

### 3.5. Transfection of Normal Human Astrocytes

**Figure 6:**
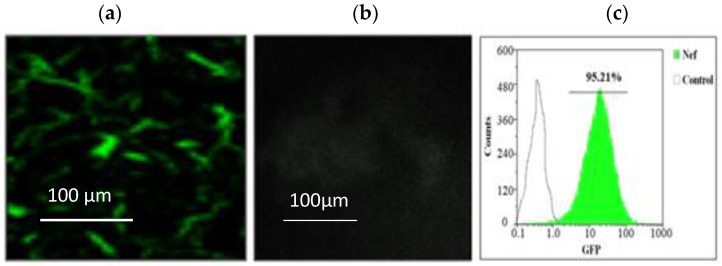
Normal human astrocytes were transfected with a pEGFP-N1-Nef plasmid expressing the Nef-GFP recombinant protein in a cell-based culture system. (**a**) The fluorescence image of astrocytes expressing the green fluorescent protein (GFP). (**b**) Untransfected NHA cells as a negative control. (**c**) The flow cytometry presents a positive expression of recombinant Nef-GFP protein in more than 95% of cells.

The constructed Nef plasmids of clinical samples, as well as subtype-specific strains, were used for transfecting the NHA after obtaining80–90% confluency. The expression of GFP was visualized by fluorescence microscopy for transfected and untransfected astrocytes. The expression level of the Nef-GFP recombinant protein was higher than 95% of transfected cells as compared to untransfected cells as the negative control, and its representative image is depicted in Figure 6.

### 3.6. Estimation of Nef Expression before and after RNA Interference

**Figure 7:**
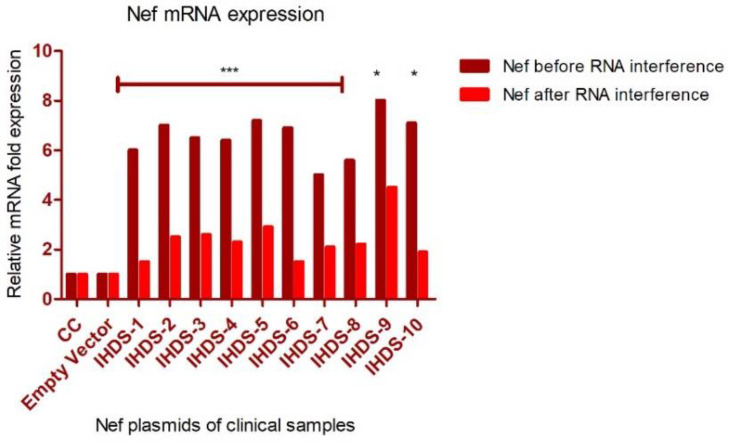
The expression level of HIV-1 Nef in NHA before and after RNA interference. We seeded 5 × 10^5^ cells in a six-well plate and transfected them with Nef plasmid (constructed using clinical samples) using Lipofectamine 3000TM.The cells were harvested and RNA was isolated to determine intracellular Nef mRNA expression at the transcription level using real-time RT-PCR. The figure depicts the fold change in mRNA expression levels relative to cell control (CC) without transfection and empty-vector-control transfected wells before and after RNA interference in NHA. The relative fold change in the Nef expression with RNA interference is statistically significant with *** *p* < 0.001 * *p* < 0.05 Dunn’s multiple comparison test. All the error bars represent standard errors, while the data shown are the mean SEM of experiments performed in triplicate.

Normal human astrocytes were transfected with the Nef plasmid of clinical samples and their expression was confirmed in the culture supernatants by quantitative real-time PCR at the transcriptional level. Another set of experiments was conducted where the transfection was blocked by RNA interference and confirmed the Nef expression by quantitative real-time PCR at the transcriptional level. There was an observance of a reduction in Nef expression to some extent, albeit not completely indicating the presence of some other mechanism responsible for reducing the expression of Nef-mRNA in the culture supernatant. The knockdown in the expression level of mRNA in astrocytes is indicated by the relative fold change at a transcriptional level before and after siRNA-Nef interference in Figure 7.

### 3.7. Expression of Cytokines, and Kynurenine Metabolites in NHA

**Figure 8:**
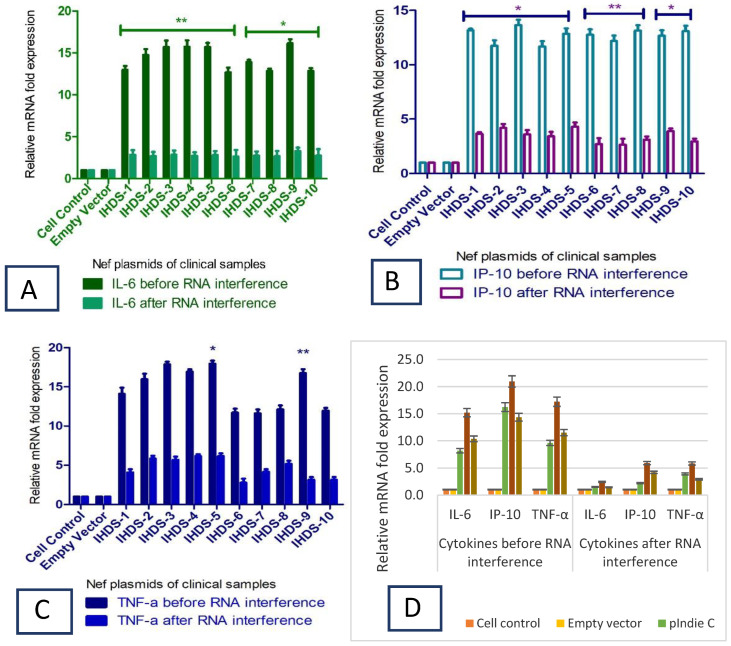
HIV-1 Nef stimulates cytokine production in normal human astrocytes. The 5 × 10^5^ cells were seeded in a six-well plate and transfected with Nef plasmid (constructed using clinical samples and subtype-specific plasmids) using Lipofectamine 3000^TM^.The cells were harvested, and RNA was isolated to determine cytokine mRNA expression at the transcription level using real-time RT-PCR. The figures indicate relative fold change in mRNA expression levels of IL-6, IP-10, and TNF-α as compared to the non-transfected-cell and empty-vector controls before and after blocking with siRNA-Nef in NHA (**A**–**C**), respectively, against Nef plasmids of clinical samples and (**D**) against subtype-specific Nef plasmids. Statistical analyses were performed using ANOVA. The relative fold change in the cytokine expression before and after blocking with siRNA-Nef is statistically significant with* *p*-value < 0.05 and ** *p* < 0.01 for all clinical samples. All the error bars represent standard errors, while the data shown are the mean SEM of experiments performed in triplicate.

Initially, we wanted to estimate the level of cytokines due to stimulation by HIV-1 Nef virotoxin in NHAs. Hence, the NHA cells were transfected with HIV Nef plasmids and the cells were harvested to measure the intracellular levels of IL6, IP-10, and TNF-α and kynurenine metabolites (IDO and HAAO) at the transcriptional level by real-time PCR. To confirm the intracellular expression levels of cytokines (IL-6, IP-10, and TNF-α) and kynurenine metabolites (IDO and HAAO), RNA isolation was conducted from the cultured cells before and after the inhibition of Nef expression using siRNA-Nef in the transfection experiment. The relative fold change in the expression was determined to estimate the impact of RNA interference. The expression data obtained using real time PCR is presented in Figure 8 (Figure 8A–C represent the expression levels of IL-6, IP-10 and TNF-α in culture supernatant after transfection with IHDS Nef specific plasmids and D represent IL-6, IP-10 and TNF-α in culture supernatant after transfection with subtype specific Nef plasmids).

### 3.8. Nef Protein Expression by Western Blotting

**Figure 9:**
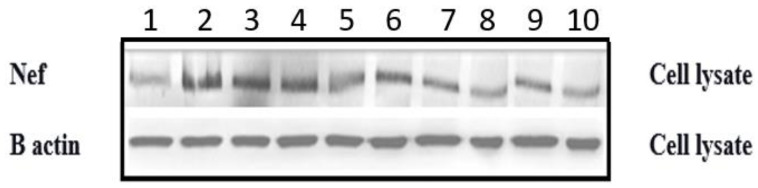
Expression of Nef protein in NHA cell culture lysates of 10 IHDS study samples (lanes 1–10). After transfection of 1 µg of Nef plasmids, the astrocytes were harvested and lysed for Western blot to measure the expression of Nef protein (M.Wt. 27 kDa). The expression of β-actin was considered an internal loading control for cell lysates (M.Wt. 42 kDa).

Normal human astrocytes were transfected with Nef plasmids of 10 clinical samples and the intracellular expression of Nef protein from cell lysates was measured by Western blot analysis. The expression of Beta-actin was considered as a loading control for cell lysates. Western blot analysis confirmed the expression of Nef by using anti-Nef and anti-beta actin antibodies, respectively. The expression of proteins is presented in Figure 9 based on data obtained from western blot experiment.

### 3.9. Downregulation of Cytokines due to siRNA-NefInterference

**Figure 10:**
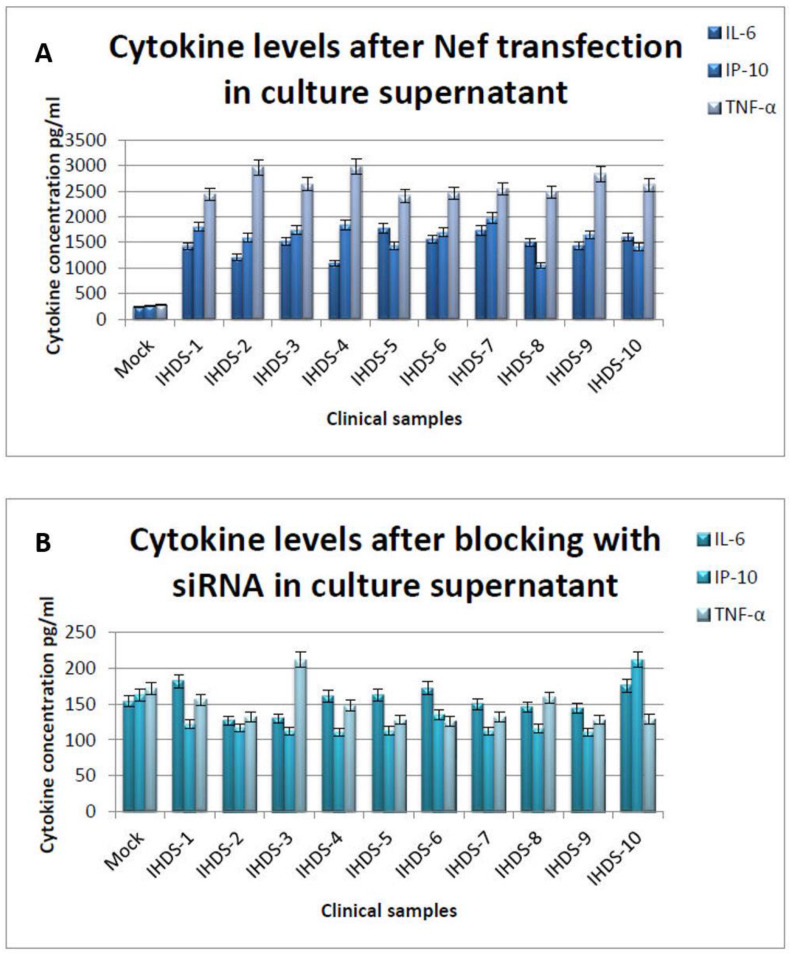
Cytokine levels were estimated using Bio-Plex Pro assay (Biorad). The 5 × 10^5^ cells were seeded in a six-well plate and transfected with Nef plasmid (constructed using clinical samples) using Lipofectamine 3000^TM^.The culture supernatants were used to determine cytokine levels at the translational level using the Bio-Plex Pro assay. (**A**,**B**) indicates cytokine levels before and after blocking with siRNA-Nef and NHA cell control. Statistical analyses were performed using one-way ANOVA.

The bioplex assay results indicate the levels of pro-inflammatory cytokines at the translational level in the astrocytes after the transfection as well as after blocking the expression of Nef with siRNA-Nef. The study results display that in the Nef plasmids of clinical samples, the expression level of the pro-inflammatory cytokines is reduced on average by five-fold in normal human astrocytes (Figure 10A,B). At the same time, the subtype-specific Nef plasmids depicted the expression levels of cytokines around five-fold, which was comparable with the expression levels of astrocytes. The expressions at translational levels were presented in triplicate and their mean values were considered for the analysis.

### 3.10. Downregulation in Kynurenine Metabolites due to RNA Interference

To obtain the impact of HIV Nef transfection on kynurenine pathway-specific metabolites at the transcriptional level, we analyzed the relative fold change in expressions of *IDO* and *HAAO* genes in NHA. The kynurenine pathway-specific gene (*IDO* and *HAAO*) expression was downregulated due to siRNA-Nef action by blocking the expression of HIV Nef clinical samples and subtype-specific plasmids. The relative fold change in mRNA expression levels was analyzed by real-time PCR and is depicted in Table 2.

### 3.11. The Level of Nitric Oxide (NO) Concentration before and after RNA Interference

**Figure 11:**
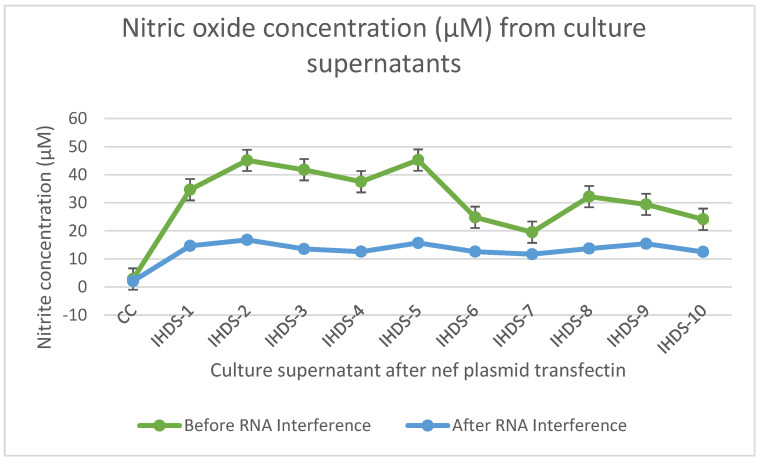
The level of nitric oxide (NO) concentration from culture supernatant was estimated before and after RNA interference. These levels are mentioned in µM concentration based on the standard curve generated using the reagents from the EZ assay nitric oxide estimation kit.

The level of NO is considered the signaling molecule for neurotransmission, inflammation, and immune responses; we estimated the NO level before and after RNA interference as depicted in Figure 11. The estimated level of NO in culture supernatant resulted in a reduction in its level after RNA interference. The statistical differences were determined by ANOVA *p* < 0.01 statistically significant.

## 4. Discussion

Neurotoxicity and cognitive impairment have been linked with inflammatory molecules produced by astrocytes expressing Nef [33,34] or virally stimulated MDM [35,36]. Furthermore, extracellular Nef released from astrocytes due to cell lysis may lead to inflammation due to the interruption of the blood-brain barrier [37] or through neurotoxicity [38]. The characterization of HIV Nef in vitro experiments for its neurotoxicity is very well reported; however, its functional aspects for developing and progressing towards HIV-associated neuropathology in the cART era need further attention. To understand this aspect, we performed cell-based experiments to determine the expression of Nef in normal human astrocytes by cloning Nef from patients with and without HIV-associated neurocognitive disorders. The in-vitro experimental results indicate that there is an upregulation in the gene expression profile of inflammatory cytokines as well as kynurenine pathway-specific metabolites, while moderate downregulation is observed after siRNA interference. At the same time, it is necessary to note that due to the ART interventions under the 90:90:90 strategies, it is less likely to obtain an in vivo expression of viral proteins in the astrocytes of HIV-positive individuals, and it is only possible where poor adherence or discontinuation of drug therapy is recorded. This becomes a limitation as against the observations found in this in vitro study.

In the present study, the intracellular expression of the HIV-1 Nef protein was found to be responsible for stimulating the secretion of cytokines and pathway-specific metabolites. The normal human astrocytes expressing Nef demonstrated elevated levels of IL-6, IP-10, and TNF-α cytokines at transcriptional and translational levels. The stimulation of cytokines might be due to variability in the expression pattern of the Nef protein, which is in concordance with the findings reported earlier [39]. Amongst all the Nef plasmids constructed from the clinical samples, only one plasmid containing a BC recombinant Nef clone (IHDS-9) demonstrated a slightly elevated level of expression than the subtype-C-specific strains of study samples, indicating its possibility in association at the level of genetic composition. Similarly, subtype-B-specific plasmid pNL4.3 also showed an elevated level of expression as compared to the subtype-C-specific plasmid, indicating the same association. The disparity in the expression levels of the Nef protein and cytokines induced in vitro experiments might have contributed to the development of neurotoxicity leading to neuropathogenesis.

The intracellular expression of Nef and damage to the integrity of the cells leading to a loss of cells was previously demonstrated with variations in the frequency of cell loss and the presence of genetic diversity of the Nef protein in different regions [40,41]. Furthermore, various pathways seem to be responsible for stimulating apoptosis in astrocytes, though the exact mechanism of cell death remains unknown. The current study showed that the intracellular expression of pro-inflammatory cytokines due to the intracellular expression of HIV Nef virotoxin and further stimulation the expression level of kynurenine pathway-specific metabolites might be an important cause of HIV Nef-induced neuropathogenesis. Additionally, the differences in the effects observed may be due to variations in the transfection efficiencies and Nef expression levels in astrocytes.

The elevated expression levels of cytokines and KP metabolites obtained for the subtype-specific plasmids, viz., subtype-B (pNL4.3) plasmid as compared to subtype-C (pIndieC) and consensus subtype-C-specific strain (pLconsNefSN) in astrocytes, confirmed the role of subtype-specific sequences. The stimulation of these cytokine-specific gene expressions matched the capacity of the Nef protein to demonstrate its potential effect on inflammation leading to the death of cells. The literary reports indicated that in various viral infections, the stimulation of cytokines has an effect on the aggregation of immune cells to the site of inflammatory infection [42] and in the brains of the simian immunodeficiency virus (SIV)-infected macaques [43]. Additionally, the data obtained from our study support the findings of stimulated cytokine expression due to the intracellular expression of the Nef protein in primary normal human astrocytes that might be associated with the Nef sequence diversity.

Even though we studied a limited number of clinical samples to assess the inflammatory potential responsible for inducing immunological markers of inflammation, we observed that there was a differential expression pattern at the transcriptional and translational levels in primary normal human astrocytes. The Nef protein from the dementia group showed an elevated expression of cytokines compared to the non-dementia group, except BC recombinant Nef protein compared to subtype-C-specific Nef protein. The presence of these differences in the expression patterns might be due to differential selection pressure acting on Nef at the cellular level as Nef is a primary target for a cellular immune response [44]. Thus, the present study supported the observation published in the literature regarding the increased neurovirulence of the Nef viral protein leading to the development of HIV-associated neurocognitive impairment in HIV-positive individuals.

Furthermore, we blocked the expression of HIV Nef using siRNA-Nef and showed its impact on the gene expression profile of kynurenine pathway-specific metabolites in primary normal human astrocytes. The fold-change reduction after blocking with siRNA-Nef was significant. Thus, our study results demonstrate the involvement of different transcription factors responsible for HIV-1 Nef-mediated cytokine production and the participation of IDO and HAAO metabolites in the kynurenine pathway. The contribution of these pathways illustrates the possible role of siRNAs targeted against viral proteins and signaling molecules. It is possible to explore this approach further to treat neuroinflammation and cognitive deficits apparent in HAND patients.

The overall relative mRNA expression of proinflammatory cytokines from the dementia group (IHDS 1–5) was higher than the non-dementia group (IHDS 6–10), indicating the impact of Nef transfection as compared to control cells without transfection. At the same time, the presence of conserved residues at P73, R78, D87, and T118 amino acids positions were essential for the downregulation of CD4 receptors, while conserved FPD amino acids at 122–124 were exposed as a loop that interacts with human thioesterase, influencing Nef-mediated endocytosis and the downregulation of CD4 and MHC1. The observations obtained from the current study correlate with previously observed findings and could be explored further with the involvement of other motifs from different genes and subtypes of HIV to correlate their role in neurotoxicity following neuroinflammation.

A differential pattern of gene expression profile might be due to the genetic diversity of HIV Nef significantly observed in normal human astrocytes (NHAs). The moderate downregulation in the expression profile might be due to siRNA-Nef, which is sequence-specific, and hence subtype-specific siRNA designing could help target the exact sequence and act along with antiretroviral therapy to control the progression of the disease. Altogether, these interpretations concur with the previous findings signifying the importance of the viral proteins responsible for contributing to HIV neuropathogenesis.

## 5. Conclusions

In conclusion, our results indicate the effect of RNA interference on the downregulation of inflammatory cytokine (IL-6, IP-10, and TNF-α) profiles as well as kynurenine pathway-specific metabolites (IDO and HAAO) moderately in NHA mimicking the setup in the brain compartment. NHAs used in this study were characterized by the elevated expressions of both proteins, and after RNA interference, the expression of inflammatory cytokines and kynurenine metabolites reduced discreetly. The genetic variability observed among the Nef isolates moderately affected the level of Nef-mediated inflammation, while subtype-specific siRNA-Nef may have less of an impact. Additionally, the levels of nitric oxide from the culture supernatant and levels of Nef expression were variable indicating the development of neurotoxicity and neuroinflammation in the astrocytes. Future studies in this field may be successful not only in the additional perception of treating neurodegenerative diseases but also in designing new strategies for potential neurological complications.

## Figures and Tables

**Table 1:** Demographic details and clinical information of the HIV-positive individuals.

IHDS * ID	Age	Sex	Mode of Transmission	IHDS * Score	CD4 Count (Cells/µL)	Viral Load (Copies/mL)	Clinical Findings
IHDS-1	45	M	Heterosexual	6.5	81	683885	Pulmonary TB, Herpes Zoster, Oral Candidiasis
IHDS-2	34	M	Heterosexual	7.5	45	325290	Herpes Zoster
IHDS-3	26	F	Heterosexual	7.0	36	2290000	Herpes Zoster
IHDS-4	40	M	Heterosexual	8.0	60	412000	Pulmonary TB
IHDS-5	35	M	Heterosexual	7.5	72	378577	Pulmonary TB, Herpes Zoster
IHDS-6	29	M	Heterosexual	11.0	198	90100	None
IHDS-7	42	M	Heterosexual	11.5	192	29386	None
IHDS-8	34	M	Heterosexual	11.0	147	57086	Herpes Zoster
IHDS-9	30	M	Heterosexual	12.0	193	68900	Herpes Zoster
IHDS-10	47	M	Heterosexual	12.0	188	74845	None

* IHDS: International HIV dementia scale.

**Table 2:** Relative fold change in the mRNA expression levels of KP metabolites *IDO* and *HAAO* in normal human astrocytes (NHAs) before and after siRNA-Nef interference. The normal human astrocyte cultures were transfected with medium alone (control) and ~1 µg/well of Nef plasmids of clinical samples and subtype-specific Nef plasmids. The Nef-specific siRNA (50 nM) was used to block before transfection, and culture supernatants were obtained to estimate *IDO* and *HAAO* in two different experiments. Data shown are the fold changes (ratios) of mRNA expression (sample versus control). The average value is considered to depict results from experiments performed in duplicate.

Sample Id. No.	Relative Fold Expression of *IDO* mRNA in NHA		Relative Fold Expression of *HAAO* mRNA in NHA	
	Before RNA Interference	After RNA Interference	Before RNA Interference	After RNA Interference
IHDS-1	3.2	1.3	4.4	1.2
IHDS-2	2.8	0.9	3.6	0.8
IHDS-3	2.0	0.5	3.1	0.4
IHDS-4	2.9	1.4	3.8	0.5
IHDS-5	2.2	1.3	3.5	0.7
IHDS-6	3.9	0.8	4.4	1.1
IHDS-7	2.2	0.2	3.5	1.2
IHDS-8	2.3	1.2	3.8	0.9
IHDS-9	6.5	1.7	5.6	1.5
IHDS-10	2.8	0.8	4.0	1.1
pIndieC	3.1	1.2	3.2	1.4
pNL 4.3	4.5	1.5	5.1	1.2
pLconsNefSN	2.9	0.5	3.1	0.5

## Data Availability

The datasets used during the present study are available from the corresponding author upon reasonable request.
